# X23D—Intraoperative 3D Lumbar Spine Shape Reconstruction Based on Sparse Multi-View X-ray Data

**DOI:** 10.3390/jimaging8100271

**Published:** 2022-10-02

**Authors:** Sascha Jecklin, Carla Jancik, Mazda Farshad, Philipp Fürnstahl, Hooman Esfandiari

**Affiliations:** 1 Research in Orthopedic Computer Science, Balgrist University Hospital, University of Zurich, 8008 Zurich, Switzerland; 2 Department of Orthopedics, Balgrist University Hospital, University of Zurich, 8008 Zurich, Switzerland

**Keywords:** 3D reconstruction, lumbar spine, digital reconstructed radiographs, convolutional neural network, artificial intelligence, computer-assisted surgery

## Abstract

Visual assessment based on intraoperative 2D X-rays remains the predominant aid for intraoperative decision-making, surgical guidance, and error prevention. However, correctly assessing the 3D shape of complex anatomies, such as the spine, based on planar fluoroscopic images remains a challenge even for experienced surgeons. This work proposes a novel deep learning-based method to intraoperatively estimate the 3D shape of patients’ lumbar vertebrae directly from sparse, multi-view X-ray data. High-quality and accurate 3D reconstructions were achieved with a learned multi-view stereo machine approach capable of incorporating the X-ray calibration parameters in the neural network. This strategy allowed a priori knowledge of the spinal shape to be acquired while preserving patient specificity and achieving a higher accuracy compared to the state of the art. Our method was trained and evaluated on 17,420 fluoroscopy images that were digitally reconstructed from the public CTSpine1K dataset. As evaluated by unseen data, we achieved an 88% average F1 score and a 71% surface score. Furthermore, by utilizing the calibration parameters of the input X-rays, our method outperformed a counterpart method in the state of the art by 22% in terms of surface score. This increase in accuracy opens new possibilities for surgical navigation and intraoperative decision-making solely based on intraoperative data, especially in surgical applications where the acquisition of 3D image data is not part of the standard clinical workflow.

## 1. Introduction

Spinal surgeries are challenging procedures due to their technical complexity and proximity to vital organs such as the spinal cord, nerves, and the aorta [1]. Several surgeon- and patient-specific factors have been reported in the prior art as the root cause for postoperative complications in such interventions [2], ranging from pedicle screw malplacement [3] to cage malpositioning [4]. In particular, the placement of pedicle screws, which is one of the most common steps in spine surgery, requires highly accurate surgical execution. Studies have shown that pedicle screw malplacement is often evaluated based on the distance between the breaching screw’s edge and the adjacent bone cortex [5]. The distances are classified into minor (<2 mm), moderate (2–4 mm), and severe (>4 mm) cases. According to literature such as [6,7], screw perforations exceeding 4 mm can cause postoperative complications. Furthermore, it has been shown that lumbar pedicle screw malplacement rates can range from 5% to 41% depending on the insertion method, the vertebral level, and the assessment protocol [3]. Pedicle screw malplacement bears the risk of neural injuries with notable negative consequences [8]. Such screw malplacements can lead to revision surgeries in up to 6% of patients [9]. Moreover, in an analysis of cage placement in posterior lumbar interbody fusion surgeries, it was identified that factors such as cage size, shape, and position can determine the lordosis in such surgeries [10]. To this end, cage malpositioning has been reported to cause nerve root compression and neurological complications [11].

Given that potential screw breaches and cage malpositionings should be ideally determined and avoided intraoperatively to prevent subsequent revision surgeries, most surgeons rely on visual assessment of multi-view (i.e., anterior–posterior and lateral views) intraoperative 2D X-rays and fluoroscopy-guided surgery as their predominant aid for surgical guidance and error prevention.

Because 2D X-ray imaging projects the 3D surgical scene onto a 2D plane, this approximation remains a poor representation of the real 3D shape of the anatomy and lacks appropriate spatial feedback. As shown in [12,13], the detection of potential pedicle screw malplacements based on planar X-ray imagery is of low accuracy (ranging from 63% to 83%) and is highly dependent on the evaluator, spinal level, and the screw material [14].

Computer-assisted surgical (CAS) navigation techniques have been introduced as means to facilitate and standardize such interventions by providing 3D intraoperative spatial guidance and were shown to result in substantially higher pedicle screw-insertion accuracy compared to free-hand and conventional 2D fluoroscopy-guided methods [3,15,16]. Furthermore, CAS technologies have also been used for cage-insertion purposes [17] and have been shown to result in improved performance [18]. The most common CAS systems still rely on preoperative 3D imaging [19] and preoperative planning, which allows for the generation of patient-specific models (bio-mechanical digital twins), which can be used as the basis for computer simulations helping to determine and optimize surgery plans. To provide intraoperative spatial guidance, these plans need to be registered to the patient’s anatomy. Although registration of preoperative data to the anatomy is a well-established concept that can be achieved through feature-based [20,21], intensity-based [22,23], or data-driven algorithms [24], it remains a challenging and sometimes error-prone process that often requires meticulous manual input [25,26]. Requiring a registration process between the preoperative and intraoperative data remains the major bottleneck in the widespread adoption of the CAS systems [22]. Additionally, due to the use of preoperatively created plans that are generated before the intervention, such systems usually fail to adapt to unforeseen intraoperative changes such as anatomical alterations and are not a suitable option in non-elective urgent surgeries where surgeons must resort to conventional, non-navigating techniques. Those and other contributing factors, such as the added cost and surgery time, have all contributed to the limited uptake of CAS systems in the community, the usage of which has been reported to be on the order of 11% of the surgeries conducted worldwide [27].

In light of the abovementioned limitations, new opportunities arise when full-fledged surgical navigation can be accomplished only based on intraoperative data and without registration. The generation of 3D representations of the anatomy instantaneously and directly in the operating room plays an essential role here. Currently, two categories of solutions exist to enable the intraoperative-only generation of 3D representations of anatomical shapes. Methods of the first category create representations by using 3D intraoperative imaging, whereas the second category consists of data-driven approaches that adapt generic atlases according to intraoperative 2D imaging.

The first category requires the use of mobile C-arm devices equipped with cone-beam computed tomography (CBCT) functionality, enabling the acquisition of 3D volumetric images during the operation. Combined with optical tracking systems, this technology allows for registration-free surgical navigation [28]. Apart from the high price of CBCT devices [16], the image acquisition process itself leads to a considerable increase in surgery time [29], and additional ionizing radiation [30].

One prominent example in the family of data-driven approaches are statistical shape models (SSMs), which capture the average shape of anatomies and their variations in atlases constructed from pre-existing databases of often healthy anatomy [31,32]. Although a variety of SSM techniques exist in the prior art, a majority of such methods still require a registration step to match the adapted atlas to the intraoperative data [33,34,35] when using them for CAS purposes. To overcome the need for performing registration, recent machine learning (ML) algorithms have been proposed that can learn to create 3D anatomical representations based on different modalities such as 2D input images [36,37,38] or ultrasound [39]. Once trained, data-driven approaches are capable of generating intraoperative 3D representations without relying on patient-specific preoperative data by fitting the trained model or atlas to fluoroscopy data [40]. A growing number of publications exist on reconstructions of patients’ 3D shapes based on 2D imaging data (i.e., [38]). Those methods process a fixed number of images from fixed poses (e.g., two bilateral X-rays taken from anterior–posterior and lateral viewpoints in [38]). Due to the end-to-end training of those networks and a limited number of pose-restricted images, a pixel-wise correspondence to the 3D shape is learned. Unseen parts of the 3D shape can be reconstructed through the underlying generalization of the network. To the best of our knowledge, most of the existing methods in this field are focused on diagnostic purposes and have not been designed and validated for intraoperative applications (i.e., CAS). Furthermore, a general issue with the data-driven methods is the limited patient specificity due to the strong prior assumptions of the models, which makes it difficult to generalize to all possible pathologies, such as bone deformities, fractures, and tumors [41]. This characteristic has been recently demonstrated on end-to-end 3D reconstruction networks that failed to sufficiently include the spatial information of the image data and tended to produce an average shape representation [42].

Multi-view fluoroscopy images are ubiquitously available in many operating rooms; however, they are currently mainly used for qualitative visual assessment purposes. In hopes of utilizing the full untapped potential of such images. In this study, we introduce a novel shape estimation approach, which we call X23D, that aims at extracting patient-specific 3D shape representations of the lumbar spine by using multi-view sparse X-ray data and the corresponding image calibration parameters as input. For this, we adapted a learnt stereo machine (LSM) [43] and trained it on a large dataset of digitally reconstructed radiographs (DRRs) generated from a computed tomography (CT) dataset. The X23D model takes 2D fluoroscopy images and their corresponding pose information as inputs and reconstructs accurate 3D shapes from a fewer number of 2D images than classic approaches [43], while conforming to the underlying geometric constraints. Because our network structure takes image projection matrices as input, anatomical shape priors can be incorporated while preserving features visible in the underlying 2D images [42]. In the context of our target application (i.e., spinal fusion surgeries), this translates into the capability of estimating high-quality 3D representations of the lumbar vertebrae in a purely intraoperative fashion and relying neither on additional imaging nor tracking apparatus for 3D reconstruction and registration purposes, respectively. This algorithm can pave the way toward a fully fetched surgical navigation solution that can provide surgery guidance tailored to intraoperative conditions. By using pedicle screw placement as an example, surgical navigation can in future work be achieved by utilizing our 3D representation with intraoperative planning to calculate ideal drilling trajectories. These plans can then, for example, be further visualized in an augmented manner, such as in [44], or be used for robotic executed or supported procedures, such as in [45].

In this paper, we lay the foundation for the design and the training of the aforementioned algorithm and evaluate its performance under different imaging conditions and against a state-of-the-art method.

## 2. Materials and Methods

**Figure 1:**
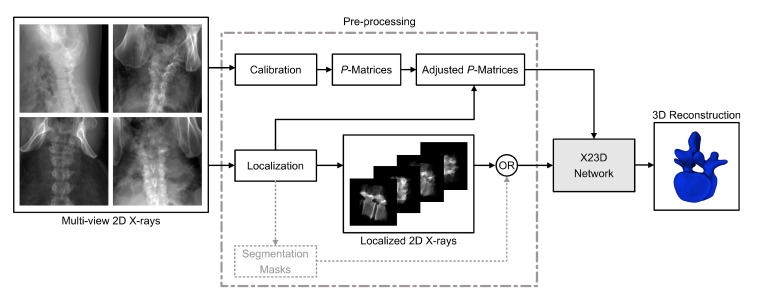
Inference pipeline of the proposed 3D shape reconstruction algorithm. Raw X-rays from multiple views are passed to a pre-processing module that estimates the imaging parameters (i.e., *P*-matrices) and crops the input images to individual lumbar vertebrae. The cropped images, along with their corresponding adjusted *P*-matrices are then passed to the X23D network to generate the 3D reconstructions. Segmentation masks can optionally be applied to the localized vertebrae before processing.

Figure 1 demonstrates the intended flow of information through our X23D algorithm during the inference stage. Assuming that our X23D algorithm is previously trained, it accepts a number of 2D X-ray images cropped down to the vertebra of interest, together with the projection matrices. For this, a pre-processing module is introduced that: (a) localizes the lumbar vertebrae in the input X-rays (and optionally segments the bone projections) and (b) calibrates the input X-rays to estimate their projection matrices including the intrinsic and the extrinsic imaging parameters. As the output of the X23D algorithm, the 3D shape representations of the individual lumbar vertebrae are generated.

The methodology section is organized as follows. First, we discuss the required pre-processing of the inputs before explaining the network architecture. Afterward, we elaborate on the data generation, including the X-ray simulation and the choice of viewing angles. In the end, we point out the train–validation–test split with its data augmentation and evaluation of the X23D’s performance in different conditions.

### 2.1. Input Data and Pre-Processing

The raw X-ray images first go through a calibration module that estimates the corresponding imaging parameters at the time of acquisition. The choice of the calibration method depends on the X23D’s use case. In a navigation setup, where the pose of the imaging device can be tracked through the use of a navigation apparatus (i.e., optical or electromagnetic tracking systems), methods such as [46] can be used. Alternatively, in setups where the use of optical tracking apparatus is rendered infeasible, precisely fabricated calibration phantoms can be used [47,48,49]. Furthermore, AI-based algorithms for C-arm calibration are currently unveiling [50,51]. In our previous work [49,51], calibration was achieved by using phantom-based and AI-based approaches, respectively, which in [49] resulted in a 0.4 mm average calibration accuracy. Therefore, the X-ray calibration module was not included in the scope of this study. After the X-ray calibration is completed for the *i*th image, the projection matrices $P_{i}$ contain the intrinsic $K_{i}$ and the extrinsic $\left[R_{i}|t_{i}\right]$ imaging parameters, all expressed in the world coordinate frame:

(1) $$P_{i}=K_{i}R_{i}\left[I|-t_{i}\right].$$

To crop the raw input X-rays in a way that they only contain the lumbar vertebra of interest, an X-ray localization module was needed during the pre-processing stage to extract the 2D bounding boxes for each vertebra. Previously, localization of vertebrae levels in X-ray images has been achieved through work such as [52,53,54,55]. Similar to X-ray calibration, X-ray localization was not covered in this study, given the existence of such methods in the prior art. A similar line of methodologies exists that can segment the projection of vertebrae on input X-rays (e.g., [56,57]). This can be fed into the X23D algorithm as an auxiliary input to assist the reconstruction task (depicted by dashed lines shown in Figure 1).

Due to the cropping introduced during the localization process, the *P*-matrices had to be adapted. Cropping to the localized vertebra results in a shift inside the image plane that can be expressed by

(2) $$\hat{P_{i}}=Q_{i}P_{i}\;,\;Q_{i}=\left[\begin{array}{ccc} 1 & 0 & -t_{i}^{x}\\ 0 & 1 & -t_{i}^{y}\\ 0 & 0 & 1 \end{array}\right],$$

where $\hat{P}$ is the adjusted *P*-matrix and $t^{x},t^{y}$ are the amount of horizontal and vertical shift introduced during the cropping stage, which are determined based on the position of the vertebral bounding box in the original full-sized X-rays.

### 2.2. X23D Network Architecture

**Figure 2:**
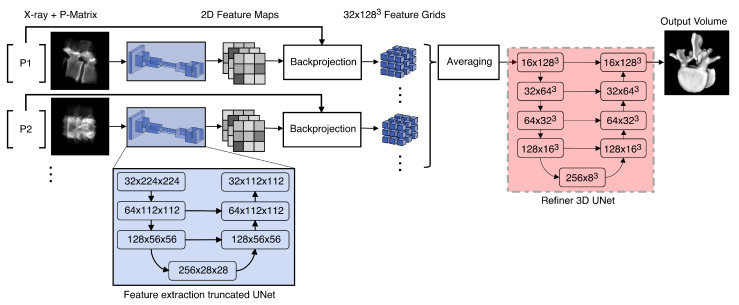
Pipeline of the proposed X23D shape reconstruction algorithm.

As its input, the X23D network accepts *i* localized X-ray images representing the anatomy from multiple views. Each image is resized to 224×224 pixels and normalized with respect to intensity to a range of [0, 1] (Figure 2). The first stage of the network is represented by a truncated UNet [58] (channels = [(1), 32, 64, 128, 256]) with skip connections that result in 32 2D feature maps $w_{i}$ of size 112×112. Those feature maps are then back-projected into the 3D space by utilizing the adjusted projection matrices $\hat{P_{i}}$, resulting each in a 128×128×128 3D feature grid $W_{i}$. The following back-projection strategy is applied to avoid ray-tracing every 2D feature in the extracted 2D feature maps $w_{i}$ from *i* viewpoints onto the associated 3D feature grids $W_{i}$. All points from the 3D feature grids $X\in W_{i}$ are projected into the 2D feature map space $x\in w_{i}$ by using $x={\hat{P}}_{i}X$. The resulting points **x** can thus be represented in a continuous 2D space, compared to the discrete locations of the 2D feature maps *w*. To assign values to the back-projected points, the discrete points of the 2D feature maps were bi-linearly interpolated. This differential back-propagation is similar to [43] and allows forward and backward passes through the neural network.

Because high-resolution feature grids are required in our application to produce high-quality 3D output shapes, the gated recurrent unit (GRU) used in [43] proved to be too memory-intensive. Hence, we replaced it with an averaging strategy, which allowed us to achieve an output resolution of 128×128×128 voxels on our available hardware (more on the used hardware in Section 2.6). The final stage was a refiner network based on a UNet architecture [58] (channels = [16, 32, 64, 128, 256]) with skip connections. The output of the finally reconstructed 3D shape was represented in the form of a 128×128×128 voxel grid. Rectified linear units have been used for all activation functions except the last one, which used sigmoid functions.

### 2.3. Data Generation and Training

Large datasets of annotated images were needed to train the X23D network (Section 2.2). Given that access to corresponding X-ray + CT scan datasets along with the annotations is a tedious undertaking, we opted for simulating the required X-ray images based on a large number of patient CT scans. To this end, we used the CTSpine1K dataset [59], which consists of 1005 CT scans of patients in DICOM format with 4712 lumbar vertebrae and their corresponding segmentation masks. The dataset was filtered according to our inclusion and exclusion criteria. Inclusion criteria were a resolution better than 1 mm in all the three axes, CT volumes bigger than 128 voxels on each axis, and the presence of all five lumbar vertebrae. Cases with pathological bone fusion (synostosis) were excluded. After filtering, the dataset consisted of 871 individual patient CT scans with a total of 4355 vertebrae from which the input–output training pairs were generated (Section 2.3.1).

#### 2.3.1. X-ray Simulation

**Figure 3:**
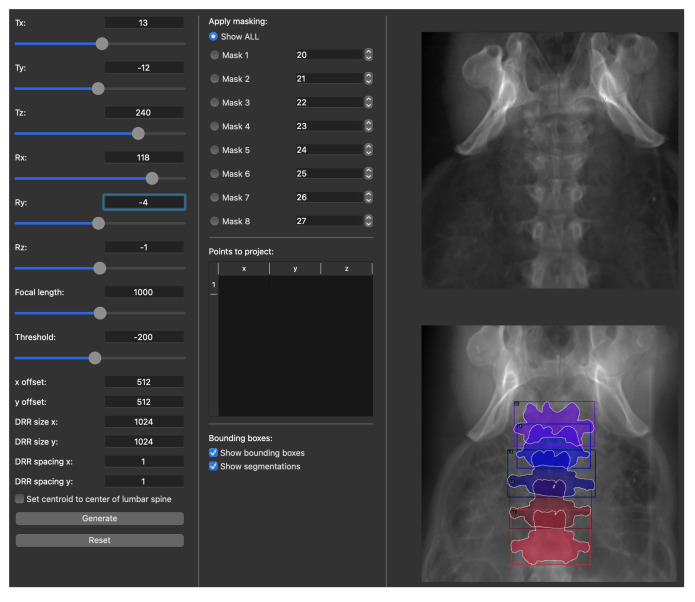
The interface of our custom-made DRR generation and annotation software. Intrinsic and extrinsic camera parameters can be adjusted on the left panel. DRR parameters such as image size and spacing can be configured. The middle part of the interface allows for applying segmentation filters on a vertebral level. Additionally, all the parameters can be passed to the back-end for batch processing of multiple CTs. The output of the software are DRRs with corresponding projection matrices, bounding boxes, and segmented areas in the COCO dataset’s format [61].

By using the collected 3D patient dataset along with the corresponding 3D segmentations, 2D X-rays and all the required 2D annotations were automatically generated in a custom-made software. The core component of the software integrates a method for generating DRR [60], which can be summarized as follows. Taking as input an object (i.e., a CT scan) with its X-ray attenuation map $A:R^{3}\to R$, our software generates a DRR image *I* through

(3) $$I\left(p\right)=\int A(T^{-1}L\left(p,s\right))ds,$$

where I(p) is the intensity of the DRR at image point *p*, $T:R^{3}\to R^{3}$ is the transformation between the object and the image plane, and L(p,s) is the ray initiating from the X-ray source and intersecting with the image plane at point *p* parameterized by *s*. The interface of our custom-developed software is shown in more details in Figure 3.

The outputs of the DRR creation and annotation software were as follows.

1. Full-size 2D DRR images generated from different view angles (more details about the view angles are provided in Section 2.3.2).
2. The corresponding *P*-matrices expressing the intrinsic and the extrinsic parameters.
3. 2D vertebral bounding boxes.
4. 2D vertebral segmentation masks.
5. Automatically localized 2D DRRs that were cropped down to a single vertebra using the bounding boxes in item 3.
6. Automatically segmented localized 2D DRRs consisting of the bone and the background classes.
7. Adjusted $\hat{P}$-matrices expressing the cropping effect.

**Figure 4:**
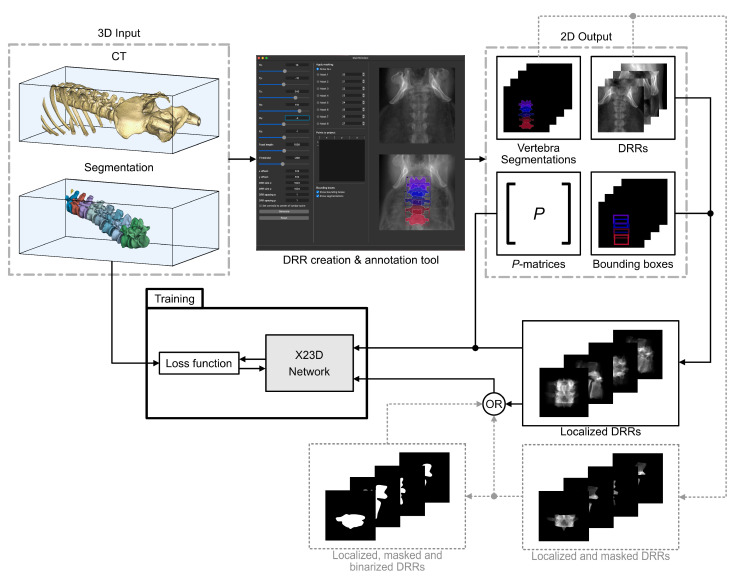
Training pipeline of the proposed X23D shape reconstruction algorithm. The 3D input, consisting of CTs and 3D segmentation masks, is passed to a DRR creation and annotation tool. The DRR tool outputs 2D DRRs, bounding boxes, segmentation masks of vertebrae, as well as the corresponding *P*-matrices. The bounding boxes and the DRRs are further processed to localized vertebrae regions before they are passed with the *P*-matrices to the training of the X23D network.

The automatically cropped DRRs and the corresponding adjusted $\hat{P}$-matrices were used to train the X23D network (Section 2.2). A detailed illustration of the training pipeline for the X23D network is provided in Figure 4.

#### 2.3.2. View Angles

The DRR approach was selected because it enabled us to simulate 2D X-rays from arbitrary viewpoints around the patient. However, poses were limited to those that can be acquired with a C-arm in an operating room, namely varying anterior–posterior, lateral, and oblique views. Therefore, four categories of viewing angles were selected:

1. AP: included three images—one strict anterior–posterior image and two images with $\pm 15^{\circ }$ deviation in the sagittal plane.
2. Lateral: included four images—two strict lateral images from each side and an additional two images with $\pm 20^{\circ }$ deviation in the coronal plane.
3. Oblique: included four images—two oblique shots with $\pm 20^{\circ }$ separation from the AP in the sagittal plane and two additional shots with $\pm 35^{\circ }$ separation from the AP in the same fashion.
4. Miscellaneous: included nine shots from poses with deviations in two planes that could not be assigned to the previously defined categories.

The 871 CTs were rotated such that each CT was in a supine position. There was intentionally no further alignment or registration to introduce additional variance within the extrinsic poses. The intrinsics *K* only depended on the DRR size, which was fixed to 224×224 pixels and the focal length. To mimic different C-arm designs, three focal lengths (850, 900, and 1000) were chosen randomly for every new DRR.

The X23D algorithm was tested on two input configurations, namely by using four or eight X-rays as inputs. According to a discussion with our surgeons, the four-view configuration was defined as the baseline, given that it was deemed clinically preferable in terms of surgery time and radiation exposure. When training the X23D network with the four-view configuration, we randomly selected one shot per view angle category (i.e., AP, lateral, oblique, and miscellaneous). For the eight-view setup, we selected the first four shots following the same approach as in the four-view setup and the second four shots were chosen randomly from all the view angle categories combined.

#### 2.3.3. Train–Validation–Test Split

The dataset was split after random shuffling into 70% training (611 patients), 20% validation (173 patients), and 10% (87 patients) test data. Given the fact that we produced 20 DRRs for every patient from all the abovementioned viewing angels, a total of 17,420 full-size DRRs were created for all the patients within our dataset (87,100 localized DRRs).

### 2.4. Performance Evaluation

In addition to using the F1 score and IoU for measuring the performance of X23D, we followed [42,62] and report the surface score, which is the harmonic mean of precision and recall at a given threshold (in our case d=1% of the side length of the reconstruction volume)

(4) $$S\left(d\right)=\frac{2\cdot Precision(d)Recall(d)}{Precision(d)+Recall(d)}\;.$$

To calculate S(d), the voxelized predictions and the ground truth were converted to meshes R and G, respectively, after which points were sampled from their surfaces. The precision and recall was then calculated as two asymmetric distances, where precision is the aggregated distances from points r∈R to g∈G

(5) $$Precision\left(d\right)=\frac{1}{n_{R}}\sum_{r\in R}\left[\min_{g\in G}∥g-r∥<d\right]$$

and recall from g∈G to r∈R

(6) $$Recall\left(d\right)=\frac{1}{n_{G}}\sum_{g\in G}\left[\min_{r\in R}∥g-r∥<d\right],$$

with *n* being the number of sampled points. Compared to the F1 score, which is a function of the overlap of target and prediction volumes, the surface score is a suitable metric for comparing fine surface details. To have a better understanding of the clinical applicability of our reconstructions and have a physical estimate of X23D’s performance, we further calculated the robust Hausdorff Distance with a 95% percentile (HD95) and the average surface distance (ASD).

Note that parts of the adjacent vertebrae and the surrounding soft tissue were present in the localized DRRs that were used to train the X23D model in Section 2.3. By using the DRR creation and annotation software (Section 2.3.1), we created two additional datasets of localized 2D DRRs that were also segmented to foreground (i.e., bone) and background (i.e., other tissues) classes. This was done for two different reasons. Counterpart 3D reconstruction models, such as Pix2Vox++ [63], generally require pre-segmented 2D inputs to reconstruct the corresponding 3D shape of the segmented area. Therefore, to have a benchmark for comparing our end-to-end algorithm to such models, we needed segmented input DRRs. Furthermore, given that the simulated DRR images that we have used to train our reconstruction network can be inevitably different in terms of image and radiological properties compared to real intraoperative X-rays, the performance of the reconstruction network may also differ when confronted with real X-ray or simulated DRR images. As a potential remedy, an investigation was performed to evaluate the feasibility of our algorithm when used on real X-ray images that have previously been segmented to foreground (bone) and background (other tissues) classes. Given that such segmentation masks will result in similar input images for our reconstruction algorithm regardless of fidelity of the underlying image (i.e., DRRs or real X-rays), we created the aforementioned segmented datasets by using DRR images. By performing this analysis, we assessed the expected performance of our reconstruction method on real X-rays that are accompanied with corresponding segmentation masks. To this end, we created two additional datasets consisting of the aforementioned segmented 2D DRRs and used them to train additional two X23D versions that could accept segmented 2D inputs, as follows.

**Figure 5:**
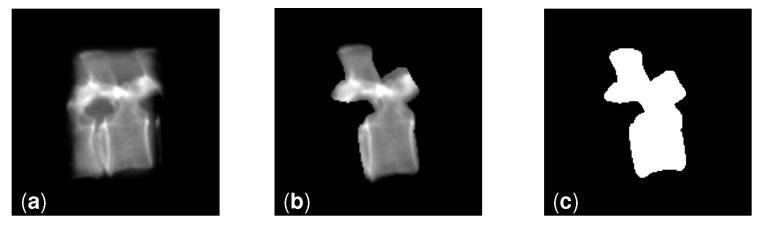
DRRs generated to train and evaluate our method showing an L1 vertebra. (**a**) Unsegmented. (**b**) Grayscale segmented using the segmentation masks in the patient CT scan. (**c**) Binarized version of (**b**).

- **Segmented grayscale:** these localized images were created by applying the 2D segmentation masks (as described in item 6 in the list above) onto the unsegmented localized images (Figure 5b).
- **Segmented binary:** these localized images were created by thresholding the segmented grayscale images (Figure 5c).

Figure 4 shows the use of the segmented grayscale and segmented binary as additional inputs for the X23D architecture, indicated by dashed lines.

Specific to the segmented binary dataset, a data augmentation process was implemented based on applying 3D elastic deformation on the underlying CT scans before creating the 2D DRR images, resulting in a doubling of the amount of 2D data (i.e., segmented binary augmented). A displacement for each voxel was interpolated from a coarse grid by using cubic B-splines with six control points. Each control point was displaced by a random amount between 0 and 18 voxels. Note that the data augmentation was only applied to the segmented binary dataset given that such augmentations would have introduced unrealistic DRR projections due to the deformation of surrounding tissue.

### 2.5. Statistical Evaluation

Apart from reporting mean and standard deviation of the results, inferential statistical tests were performed to compare the results of our algorithm to the ones from the benchmark method (i.e., Pix2Vox++). Furthermore, we ran statistical testing to determine whether or not there was a statistically significant difference between the reconstructions achieved by segmented versus unsegmented 2D input. After initial investigation of the reconstruction results, we determined that they do not follow a normal distribution; thus, we applied a Mann–Whitney U tests for all of our inferential statistics. An α-value of 0.05 was selected and Bonferroni-corrected.

### 2.6. Coding Platform, Hardware and Training Hyperparameters

For the DRR creation and annotation software, the Insight Toolkit (ITK) framework [64] and the SimpleITK framework [65] were used. In addition, PySide6 and QT were used to create the interface. The elastic deformations were applied by using TorchIO’s [66] *RandomElasticDeformation* function. The network was built with PyTorch [67] and trained on a GPU cluster consisting of multiple Nvidia RTX 2080 Ti with 11 GB VRAM and Nvidia GTX 1080 TI with 11 GB VRAM. Training of the networks required between 30 and 40 epochs with a learning rate of $10^{-4}$. Early stopping was applied to regularize the training. Due to the large output volume size of 128×128×128 voxels, we leveraged multiple GPUs and PyTorch Lightning’s [68] accumulated gradients, which resulted in an effective batch size of 16. In the eight-view configuration, training on eight GPUs lasted approximately seven days.

For the calculation of the metrics we used Deepmind’s surface-distance repository (https://github.com/deepmind/surface-distance accessed on 5 September 2022). For the statistical evaluation SciPy [69] and R have been used.

## 3. Results

Table 1 reports the performance of our proposed method on unseen data (unseen patients and unseen DRRs) by using the F1, IoU, and surface scores. This table also reflects on the performance of our algorithm with pre-segmented input X-rays. Additionally, we have compared the results of X23D to the results achieved by the Pix2Vox++ network.

On unsegmented vertebrae and with four views, our method outperformed the four- and eight-view versions of Pix2Vox++, with a 0.88 average F1 score. Our network’s performance increased slightly when pre-segmentation was available, which was indicated by an increase in the F1 score from 0.88 to 0.89 in the four-view setup. A more notable performance difference can be seen in the surface scores, where our network performed 22% better than Pix2Vox++.

According to the statistical test carried out, all of our methods perform significantly better than the benchmark method Pix2Vox++ regardless of the number of views. Our main approach with four unsegmented views does not perform significantly differently from the segmented binary approach in terms of F1 score or surface score. Additionally, there is no significant difference comparing the surface score results of our main approach (unsegmented four-view) to the augmented segmented binary approach.

Table 2 shows the results of the evaluation of the unsegmented four-view approach from Table 1 broken down to the vertebrae level L1 to L5. The F1 scores deviated only slightly from the average across all levels. L1 and L2 resulted in similar performances with only slightly above average F1 scores. However, the surface scores of both were 5% better than average. Performance drops gradually towards L5, with the surface score for L4 being 3% and L5 15% below the average.

**Figure 6:**
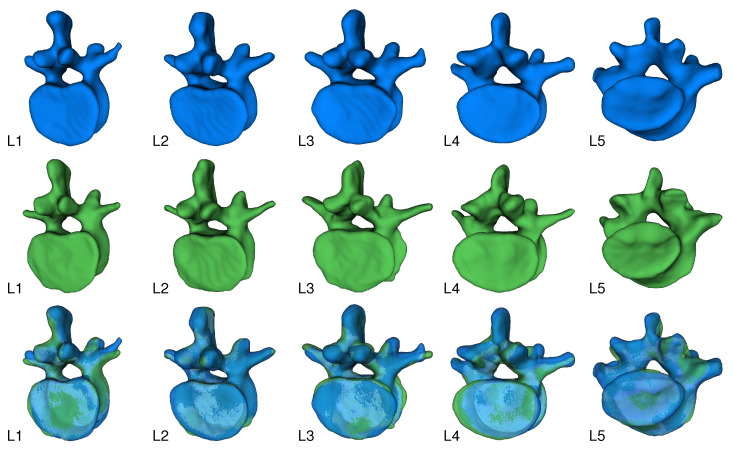
Visualized output of the X23D algorithm with unseen input. The upper row shows the predictions for L1 to L5 using four unsegmented images as input. The middle row shows the corresponding ground truth. The bottom row shows an overlay of prediction and ground truth.

**Figure 7:**
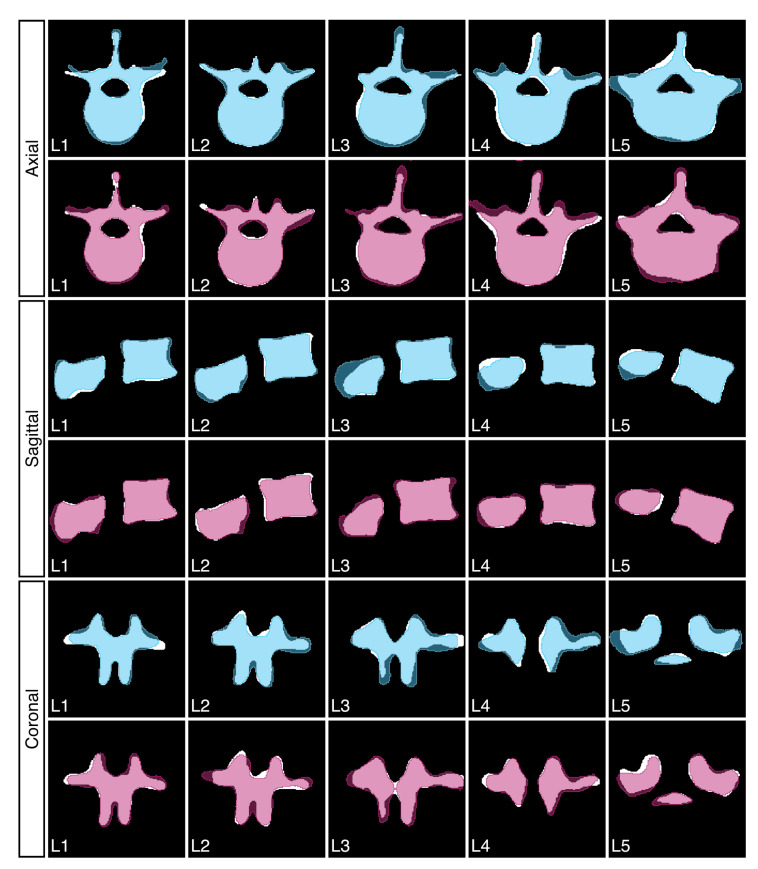
The 2D slices extracted from the results reported in Figure 6. The ground truth is depicted in white. Blue shows results using four unsegmented unseen views as input. Magenta shows results using four unseen grayscale segmented views.

**Figure 8:**
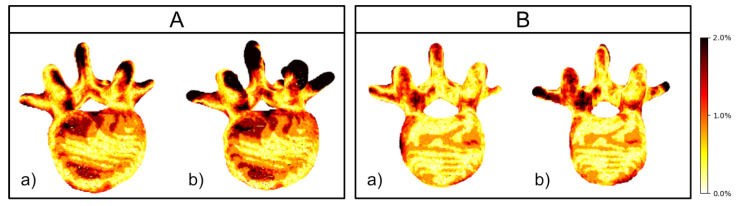
Visualization of the surface score on two X23D reconstructions each using four-view unseen and unsegmented inputs. Distance is expressed in color with distances bigger than 2% of the volume side length being black. (**a**) Visualization of point distances to compute the precision. (**a**) Distances measured from the target to the closest predicted point. (**b**) Visualization of point distances to compute the recall. (**b**) The distance from the prediction to the closest target point. Darker areas in (**a**) (precision) indicate areas that were reconstructed but are not present in the target (as seen in the spinous process and transverse area of (**A**). Darker areas in (**b**) (recall) indicate areas that are missing in the prediction. All points with distances bigger than a 1% threshold (defined in Section 2.4), ranging from red to black, contributed to lowering either recall or precision. The spinal canal was reconstructed correctly in both examples despite not being visible in input images. (**B**) Overall good precision, recall and, hence, surface score.

Figure 6 shows sample results of our reconstruction algorithm trained on four views of unsegmented and grayscale segmented data, respectively. Slices of the axial and sagittal planes from the 3D reconstructions in Figure 6 are shown in Figure 7 for better comparison. In Figure 8, visualizations of precision and recall are shown with a threshold of 2% for two unseen test cases.

The reconstruction of a vertebra, including loading of the images, took on average 4.2 s for the four-view arrangement and 7.9 s for the eight-view arrangement. These values were measured during the inference of 100 vertebrae on a workstation with NVIDIA RTX 2080 Super and an i7-8700 CPU @ 3.20 GHz.

## 4. Discussion

This study showed that accurate patient-specific 3D representations of the spine anatomy can be generated from a sparse set of four X-ray images. We introduced X23D as a complete pipeline for data processing, network training and inference, the performance of which was tested on unseen data. Compared to the state-of-the-art 3D reconstruction algorithms such as Pix2Vox++, our method achieved a 22% higher surface score, which can be attributed to the exploitation of the spatial information present in the projection matrices. Compared to the status quo in conventional spinal surgeries where on average 54 X-ray views are acquired [70], and CBCT-based operations where 100–120 fluoroscopy views are needed [29], our method required substantially fewer X-rays to produce accurate 3D representations.

The X23D algorithm achieved high-quality solutions even in cases where no pre-segmentation of the input X-rays was available. In such cases, the 3D shape reconstruction accuracy of X23D was 0.88 in a four-view setup as assessed by the F1 score. The only principal requirement was the availability of localization of individual vertebral levels on the input X-rays, which has been presented in studies such as [54]. In cases where a pre-segmentation of vertebrae were present, we demonstrated a slight statistical improvement in X23D’s reconstructions, where the F1 score and the surface score were improved by an additional 1% and 3%, respectively. The segmented binary augmented approach did not perform significantly different than our main approach (unsegmented four-views). This speaks for the application of our method on real X-ray images. The result for the individual levels shows a clear drop in performance for L5. This can be attributed to the fact that L1 to L4 look more similar than L5. More training data could allow training of models for individual vertebral levels, which could increase the overall performance.

Despite higher F1 scores, this study was not able to show a significant improvement of the eight-view variants over their four-view variants in terms of surface score. One potential reason might be that the current network architecture did not have enough capacity to reveal its full potential given more views. This might be connected with the simplistic merging stage chosen in the herein implementation of the network to reduce the memory consumption to an acceptable level. Alternative architectures such as attention mechanisms will be evaluated in future work. Upon close visual inspection of the precision-recall plots (as example shown in Figure 8), anatomical regions of high clinical importance such as the spinal canal, vertebra body, and pedicles were areas with high reconstruction precision, which is especially important for applications such as pedicle screw placement surgery.

The inference time of 4.2 s for the four-view and 7.9 s for the eight-view constellation, respectively, show the potential of our algorithm for intraoperative use cases. In comparison to prior art on spinal reconstruction were computation time off approximately four seconds has been reported (e.g., [35]), our approach has a rather comparable computation time. Note that neither the network nor the data loader are optimized for speed yet. Therefore, further speed gains are expected to enable intraoperative lumbar spine reconstruction in a few seconds.

This study had limitations. Although stemming from real patient CT scans, the X-ray images used for training and testing the X23D algorithm were simulated by using a custom-made software to streamline the data collection, annotation and curation in light of the practical hurdles along the collection of paired real CT and X-ray datasets. Prior work, such as [71,72], show that clinically feasible simulations of X-rays can be achieved by more advanced simulation methods. Such X-ray simulation algorithms will be implemented into the X23D pipeline in the future phases of this project. Nevertheless, in an effort to demonstrate the potential of the current X23D network when faced with real X-ray data, we explored its reconstruction accuracy when 2D segmentation masks are available. This mimics a scenario when a real X-ray is first segmented and later fed to the X23D algorithm. By performing such additional evaluations, we showed that our approach can be extended to a segmentation and reconstruction pipeline that can achieve similar 3D reconstruction performance on real X-rays. In the future, an in vivo data collection study will allow us to collect real X-rays with corresponding calibration data by using our calibration phantom. The reconstruction accuracy depends on the quality of the calibration. However, a comprehensive sensitivity analysis of the calibration parameters was beyond the scope of this work, but is planned for the future. Nevertheless, we performed a preliminary sensitivity analysis to determine the applicability of our method. Perturbations of up to 50 mm were applied separately to the focal length parameters of either anterior–posterior or lateral images. For perturbed lateral or anterior–posterior images, a monotonous performance decrease in the F1 score of up to 3% and 2% respectively, was observed. The surface scored decreased monotonously by up to 7% for lateral and 5% for anterior–posterior perturbed images.

## 5. Conclusions

In this work, an end-to-end approach to the 3D reconstruction of vertebra of the lumbar spine was presented. Our method requires only sparse 2D fluoroscopic data to achieve accurate reconstructions, making it a prime candidate for intraoperative applications. This was achieved by explicitly incorporating the calibration parameters as network inputs, which can be considered as one of our key contributions. We see big potential in using our method as a component in the next generation of computer-aided interventions and surgical navigation, especially in surgical applications in which the acquisition of 3D image data is not part of the clinical workflow.

## Figures and Tables

**Table 1:** Performance of our X23D algorithm. Best results presented in bold font. Quantitative comparisons are provided when the X23D network was trained on segmented and unsegmented 2D inputs. Additionally, the performance of X23D is compared to the results achieved through Pix2Vox++ trained on segmented inputs.

	Unsegmented		Segmented				Pix2Vox++ (Seg.)	
			Grayscale		Binary	Binary-Aug.		
**#Views**	**4**	**8**	**4**	**8**	**4**	**4**	**4**	**8**
F1 score	0.88 ± 0.03	0.89 ± 0.04	0.89 ± 0.03	**0.91** ± 0.02	0.90 ± 0.02	**0.91** ± 0.02	0.85 ± 0.05	0.86 ± 0.06
Surface score	0.71 ± 0.11	0.67 ± 0.12	**0.73** ± 0.10	0.69 ± 0.10	0.70 ± 0.10	0.72 ± 0.09	0.58 ± 0.12	0.58 ± 0.12
IoU	0.89 ± 0.03	0.89 ± 0.03	0.90 ± 0.02	**0.91** ± 0.02	0.90 ± 0.03	**0.91** ± 0.02	0.86 ± 0.04	0.87 ± 0.04
HD95 (mm)	2.83 ± 1.72	3.21 ± 1.84	2.31 ± 0.94	3.79 ± 4.51	2.43 ± 0.92	**2.20** ± 0.66	3.74 ± 2.01	3.76 ± 1.90
ASD (mm)	0.73 ± 0.36	0.84 ± 0.43	**0.67** ± 0.21	1.02 ± 0.66	0.73± 0.20	0.73 ± 0.18	1.01 ± 0.44	0.99 ± 0.39

**Table 2:** Performance of our X23D algorithm evaluated on individual vertebral levels by using four unsegmented views as input. Best results presented in bold font.

	4-Views				
	**L1**	**L2**	**L3**	**L4**	**L5**
F1 score	0.89 ± 0.03	**0.90** ± 0.02	0.89 ± 0.03	0.88 ± 0.03	0.86 ± 0.04
Surface score	0.75 ± 0.09	**0.76** ± 0.09	0.73 ± 0.09	0.69 ± 0.11	0.62 ± 0.11
IoU	**0.90** ± 0.02	**0.90** ± 0.02	0.89 ± 0.02	0.89 ± 0.03	0.87 ± 0.03
HD95 (mm)	**2.39** ± 1.05	**2.39** ± 1.12	2.87 ± 1.79	3.10 ± 2.46	3.38 ± 1.60
ASD (mm)	0.64 ± 0.18	**0.62** ± 0.16	0.67 ± 0.16	0.81 ± 0.65	0.90 ± 0.30

## Data Availability

The code presented in this study is available on request from the corresponding author. The code is not publicly available due to a pending patent application. The described dataset is available here: https://github.com/MIRACLE-Center/CTSpine1K (accessed on 13 May 2022).

## Abbreviations

The following abbreviations are used in this manuscript:

ASD	Average Surface Distance
CAS	Computer-Assisted Surgical
CBCT	Cone-Beam Computed Tomography
CT	Computed Tomography
DRR	Digitally Reconstructed Radiograph
GRU	Gated Recurrent Unit
ITK	Insight Toolkit
LSM	Learnt Stereo Machines
ML	Machine Learning
SSM	Statistical Shape Model
